# Short-Term Efficacy of Two-Step Treatment of Retinopathy of Prematurity in a Japanese Cohort: Anti-VEGF Therapy Followed by Routine Laser Photocoagulation

**DOI:** 10.3390/jcm14197094

**Published:** 2025-10-08

**Authors:** Shimpei Oba, Tatsunori Kiriishi, Masatoshi Omi, Yuki Hattori, Hidetsugu Mori, Masayuki Ohnaka, Takeshi Hoshino, Haruhiko Yamada, Hisanori Imai

**Affiliations:** Department of Ophthalmology, Kansai Medical University, 2-5-1 Shin-machi, Hirakata 573-1191, Japan; oba.shi@kmu.ac.jp (S.O.); kiriishi.tat@kmu.ac.jp (T.K.); omi.mas@kmu.ac.jp (M.O.); hattori.yuk@kmu.ac.jp (Y.H.); mori.hid@kmu.ac.jp (H.M.); t.hoshino127@gmail.com (T.H.);

**Keywords:** retinopathy of prematurity, anti-VEGF, laser photocoagulation, bevacizumab, ranibizumab

## Abstract

**Objective**: The aim of this study was to assess the efficacy of laser photocoagulation (LPC) combined with anti-vascular endothelial growth factor (VEGF), in comparison with routine LPC monotherapy, in the treatment of retinopathy of prematurity (ROP). **Methods**: This retrospective study included data from 142 eyes treated according to the standard criteria of the Early Treatment for Retinopathy of Prematurity (ETROP). Group A patients had received LPC alone, and Group B had received anti-VEGF therapy followed by routine LPC. Group B was further categorized into two subgroups: Groups B1 and B2 had received bevacizumab and ranibizumab, respectively. Data collected included ROP stage, gestational week, postmenstrual week, birth weight, number of laser spots and sessions. **Results:** Group B required significantly fewer laser spots than was the case with Group A (Group A: 583.0 ± 350.72, Group B: 274.9 ± 124.77, *p* < 0.0001). The number of LPC sessions differed significantly between the groups (Group A: 1.8 ± 1.28, Group B: 1.2 ± 0.45, *p* = 0.0003). **Conclusions:** Combining anti-VEGF therapy with routine LPC reduced the number of laser spots required. This approach offers an effective treatment strategy for managing severe ROP, potentially reducing long-term complications associated with extensive laser use.

## 1. Introduction

Retinopathy of prematurity (ROP) was first described as retrolental fibroplasia (RLF) in 1942 [1]; the term “ROP” was later adopted in 1951 [2]. ROP is characterized by biphasic pathophysiology: the initial phase involves oxygen-induced suppression of retinal vascular development, followed by a second phase marked by hypoxia-driven production of vascular endothelial growth factor (VEGF) and pathological neovascularization. Severe vascular underdevelopment and neovascularization can result in complications, such as vitreous hemorrhage or tractional retinal detachment, leading to significant visual impairment. In 1968, Nagata et al. introduced xenon photocoagulation to inhibit the progression of ROP [3]. Subsequently, laser photocoagulation (LPC) became the standard treatment. LPC, as an established first-line therapy, ablates the peripheral retina, thereby reducing VEGF production in hypoxic regions. In a 2004 study titled Early Treatment for Retinopathy of Prematurity (ETROP), it was shown that early diagnosis and timely LPC application could improve the prognosis of severe ROP [4]. However, in more recent studies, LPC has been associated with adverse outcomes, such as high myopia and restricted visual fields [5,6], limiting its ability to fully meet therapeutic needs.

The discovery of VEGF as a key factor in tumor angiogenesis in 1989 led to the development of anti-VEGF agents [7], which have since been applied to various neovascular diseases, including ROP. In 2011, a study titled Bevacizumab Eliminates the Angiogenic Threat of ROP (BEAT-ROP) involved 150 infants (300 eyes) with stage 3+ ROP in Zone I or posterior Zone II. The researchers compared the effectiveness of intravitreal bevacizumab (IVB, 0.625 mg) monotherapy to that of LPC. At 54 weeks postmenstrual age, IVB significantly reduced recurrence rates in Zone I ROP (4% vs. 22%; *p* = 0.003), demonstrating superior efficacy over LPC in this zone although comparable in Zone II [8]. In the 2019 RAINBOW study, a multinational phase III randomized trial involving 225 infants with ROP in Zones I and II, the efficacy of intravitreal ranibizumab (IVR) was evaluated at doses of 0.1 and 0.2 mg, compared with outcomes of LPC. The IVR 0.2 mg group achieved a treatment success rate of 80.0%, compared to 66.2% in the LPC group, indicating non-inferiority and potential superiority of IVR treatment [9]. More recently, in the FIREFLEYE trial, the treatment outcomes of intravitreal aflibercept (IVA, 0.4 mg) were assessed in 113 infants with ROP; treatment with IVA was compared with LPC in a 2:1 randomization. At 24 weeks, IVA achieved a favorable outcome rate of 85.5%, compared to 82.1% in the LPC group, meeting predefined criteria for non-inferiority and suggesting comparable efficacy [10]. Based on these findings, anti-VEGF therapy has become a key treatment option for ROP. Nevertheless, anti-VEGF agents differ in potency, binding affinity to VEGF family members, and intraocular half-life, resulting in varying dosing regimens [11]. Systemic exposure to these agents can also affect organ development, raising safety concerns, especially in neonates [12,13]. Although anti-VEGF therapy has a lesser impact on ocular growth and myopia compared to LPC, it has been associated with chronic vascular arrest (also known as persistent vascular stasis), which can increase the risk of ROP recurrence and the need for retreatment [14,15]. In this context, determining how to complete treatment while minimizing the number of LPC and anti-VEGF interventions remains an important clinical question. Therefore, there is a continued need for new therapeutic strategies for ROP. However, there are few reports that have examined the efficacy of a combined treatment using both LPC and anti-VEGF therapy.

The aim of the present study was to evaluate whether two-step therapy combining anti-VEGF therapy with routine LPC offers enhanced treatment efficacy compared to LPC monotherapy in a Japanese cohort. By comparing these treatment approaches, we sought to identify optimal strategies to maximize therapeutic outcomes in the management of ROP.

## 2. Materials and Methods

This retrospective study included data from 71 pediatric patients with severe ROP (dataset of 142 eyes), treated according to the ETROP criteria [16], with a 1-year follow-up period. The treatment methods consisted of LPC monotherapy prior to 2016, and combination therapy with anti-VEGF therapy and routine LPC from 2016 onward. As anti-VEGF therapeutics, bevacizumab was used from 2016 to 2022 and ranibizumab after 2022. The study population was categorized into two primary groups: Groups A (before 2016), eyes treated with LPC monotherapy for severe ROP; Group B (2016–2024), eyes treated with anti-VEGF therapy, followed by routine LPC. In Group B, LPC was performed 4 weeks after the anti-VEGF therapy. Group B was further categorized into two subgroups: Group B1 (2016–2022): eyes treated with IVB; Group B2 (2022–2024): eyes treated with IVR. The study was approved by the Ethics Committee of Kansai Medical University Hospital (Approval No. 2024283, 7 February 2025), and was conducted in accordance with the principles of the Declaration of Helsinki. For cases involving intravitreal bevacizumab (IVB), oral informed consent for off-label use was obtained from the parents of the pediatric patients. The following clinical data were retrospectively collected from medical records using the established ETROP ROP staging classification for treatment interventions [16]: ROP stage classification and postmenstrual age (PMA) at the time of intervention, gestational age (GA), birth weight (BW), number of LPC spots, number of LPC sessions, and requirement for vitrectomy.

### 2.1. Laser Photocoagulation

LPC was performed in the Neonatal Intensive Care Unit (NICU) under sedation provided by a pediatrician. A monocular indirect ophthalmoscope (BS-IILED, Neitz Instruments Co., Ltd., Tokyo, Japan) equipped with a 20-diopter lens (Volk Optical Inc., Mentor, OH, USA) and an LPC attachment (Nidek Co., Ltd., Gamagori, Aichi, Japan) was used for the procedure. Laser treatment was applied via 200-µm diameter spots at a power of 200 mW for 0.2 s. The laser spots were spaced at intervals of 1–2 spot diameters within the avascular retinal area, targeting regions one optic disc diameter from the edge of the vascularized retina. When performing peripheral LPC, scleral indentation was performed by an assistant.

### 2.2. Intravitreal Injection of Anti-VEGF Agents

Intravitreal injections of anti-VEGF agents were performed in the operating room under sedation by an anesthesiologist, with pediatricians present to monitor the procedure. After sterilizing the ocular surface and conjunctival sac with povidone-iodine, a skilled ophthalmologist administered 0.01 mL of bevacizumab (Avastin^®^, Roche, Basel, Switzerland) or 0.01 mL of ranibizumab (Lucentis^®^, Novartis, Basel, Switzerland) using a 30-gauge needle inserted 1 mm posterior to the corneal limbus.

### 2.3. Statistical Analysis

Statistical analysis was performed using JMP® Pro 17.2.0 (SAS Institute Inc., Cary, NC, USA). Normality of continuous variables was assessed using the Shapiro–Wilk test. All variables showed non-normal distribution (*p* < 0.05), therefore non-parametric tests were applied. The chi-square test was applied to compare treatment groups in terms of ROP stage and the need for vitrectomy. The Wilcoxon rank-sum test was used to compare PMA, GA, BW, the number of LPC spots, and the number of LPC sessions between the two groups. The Wilcoxon rank-sum test was employed to compare three groups, followed by Steel-Dwass tests. We performed a multivariate analysis with LPC spot number as the dependent variable and BW, GA, PMA, treatment type (Group A or B), and ROP severity based on zone classification as explanatory variables to identify factors associated with LPC spot number. A *p*-value < 0.05 was considered statistically significant.

## 3. Results

**Figure 1:**
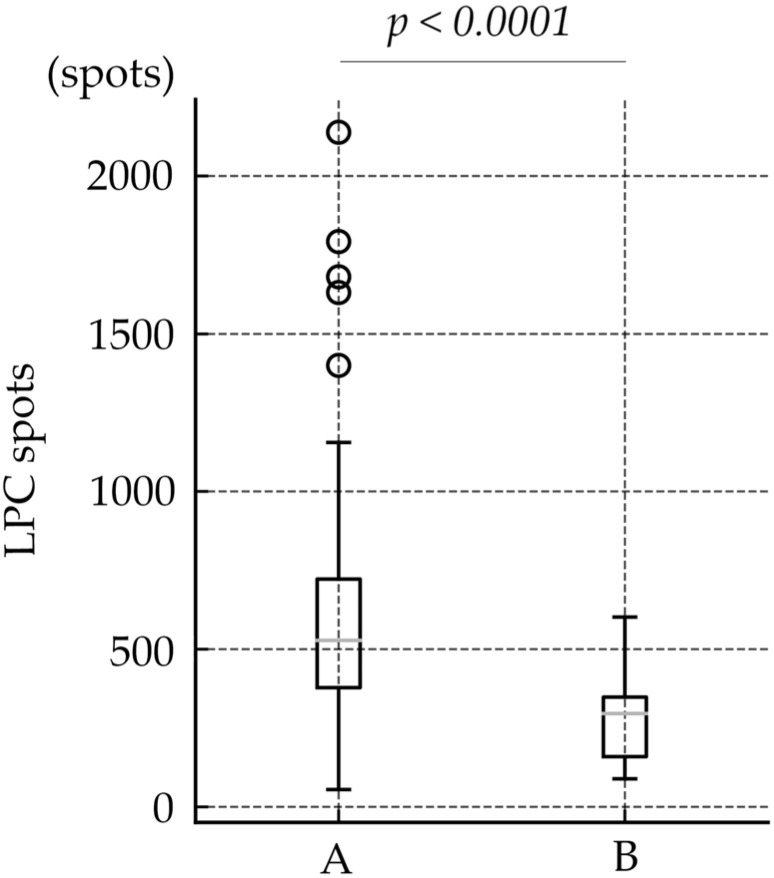
Comparison of LPC Spot Numbers Between Group A and Group B. The mean number of LPC spots was 583.0 ± 350.72 in Group A and 274.9 ± 124.77 in Group B. Group B required significantly fewer laser spots than Group A (Wilcoxon rank-sum test, *p* < 0.0001).

**Figure 2:**
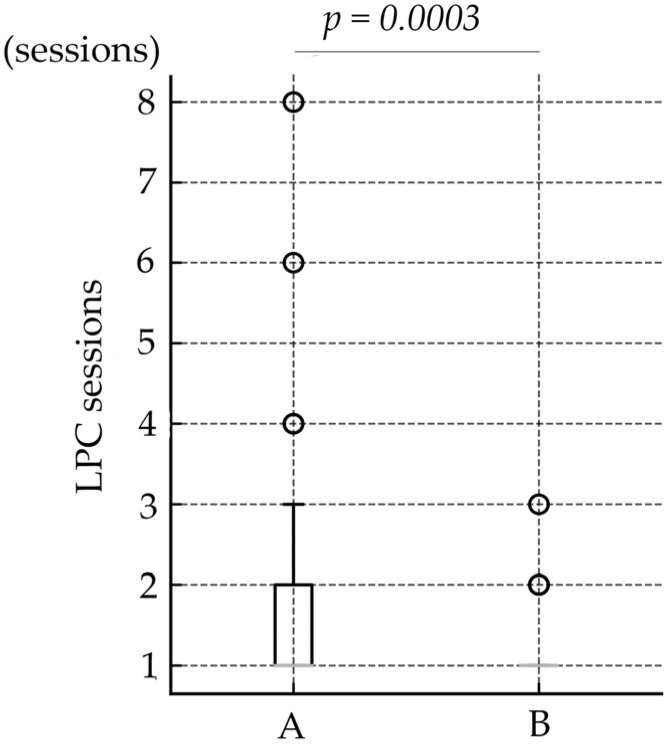
Comparison of LPC sessions between Group A and Group B. The mean number of LPC sessions was 1.8 ± 1.28 in Group A and 1.2 ± 0.45 in Group B. Group B required significantly fewer sessions than Group A (Wilcoxon rank-sum test, *p* = 0.0003).

Patient demographic data are presented in Table 1. Data from a total of 142 eyes were included in the study, categorized into Groups A (49 patients, 98 eyes) and B (22 patients, 44 eyes), with Group B further subdivided into Groups B1 (14 patients, 28 eyes) and B2 (8 patients, 16 eyes). The mean BW (g) was 720.3 ± 188.39, 652.3 ± 133.49, and 699.2 ± 175.02, in Group A, Group B, and total, respectively. This difference was not statistically significant between Group A and B (Wilcoxon rank-sum test, *p* = 0.0504). The mean GA (weeks) was 26.0 ± 1.67, 25.0 ± 1.62, and 25.6 ± 1.69, in Group A, Group B, and total, respectively. A significant difference was observed between Group A and B (Wilcoxon rank-sum test, *p* = 0.0023). The mean PMA (weeks) at the time of intervention were 33.5 ± 2.09, 33.4 ± 2.11, 33.5 ± 2.15, in Group A, Group B, and total, respectively. PMA values did not differ between Group A and B (*p* = 0.96). At the time of intervention, eyes were diagnosed as follows in Group A: Zone 2, Stage 2+ in 64 eyes; Zone 2, Stage 3+ in 3 eyes; Zone 1, Stage 3 in 1 eye; Zone 1, Stage 1+ in 8 eyes; Zone 1, Stage 2+ in 8 eyes; and Zone 1, Stage 3+ in 14 eyes. In Group B, the distribution was as follows: Zone 2, Stage 2+ in 14 eyes; Zone 2, Stage 3+ in 0 eyes; Zone 1, Stage 3 in 1 eye; Zone 1, Stage 1+ in 7 eyes; Zone 1, Stage 2+ in 19 eyes; and Zone 1, Stage 3+ in 3 eyes. The mean number of LPC spots (spots) was 583.0 ± 350.72, 274.9 ± 124.77, and 487.6 ± 330.20, in Group A, Group B, and total. Eyes in Group B required significantly fewer laser spots than did those in Group A (Wilcoxon rank-sum test, *p* < 0.0001) (Figure 1). The number of LPC sessions were 1.8 ± 1.28, 1.2 ± 0.45, and 1.6 ± 1.12, in Group A, Group B, and total. The number of LPC sessions differed significantly between Group A and B (Wilcoxon rank-sum test, *p* = 0.0003) (Figure 2). Regarding the need for vitrectomy, three eyes in Group A required vitrectomy, whereas no eyes in Group B required the procedure, with no significant difference between the groups (chi-square test, *p* = 0.59). During the follow-up period, no cases of disease recurrence were observed in either group. The subdivided analysis between B1 and B2 group showed no significant differences in BW, GA, PMA, number of LPC spots, and number of LPC sessions.

Furthermore, multivariate analysis revealed that BW(*p* = 0.0017), zone classification (*p* = 0.0376), and treatment group (*p* < 0.0001) remained as significant determinants of LPC spot number (Table 2).

The severity of ROP by treatment era, especially in terms of vascular extension, was compared between Group A and Group B, with zone classification used as an indicator (Table 3). In Group A, Zone 1 in 31 eyes and Zone 2 in 67 eyes, on the other hand, in Group B, Zone 1 in 30 eyes and Zone 2 in 14 eyes. The difference in zone distribution was statistically significant (chi-square test, *p* < 0.0001).

## 4. Discussion

In clinical practice, anti-VEGF therapy is increasingly viewed as a first-line treatment for ROP [17]. However, disease recurrence remains a significant concern with monotherapy [18]. Reported recurrence rates vary among anti-VEGF agents: IVB has shown recurrence rates ranging from approximately 4% to 16% depending on study design, follow-up duration, and population [8,19,20]. The recurrence rates of IVR range from 15% to 63% [9,21,22,23]. IVA has been reported to have a recurrence rate of 5% to 22% based on pooled analysis from the FIREFLEYE trial [10,24]. In a retrospective study of aggressive ROP, IVR showed significantly higher reactivation rates [92.7% compared to 52.8% with IVB (*p* < 0.001)], and reactivation occurred earlier (mean 7.7 vs. 12.8 weeks for IVB, *p* < 0.001) [25]. In a large multicenter cohort study (ROPIC), retreatment rates of 25.0% and 17.6% were reported for IVR (including biosimilar) and IVB, respectively, with IVB showing a significantly delayed recurrence, although the difference was not statistically significant (*p* = 0.1) [26]. In another retrospective study, in Japan, investigators reported that IVR-treated eyes required additional laser significantly more often (13 vs. 1 eye in IVB group), with mean time-to-recurrence of 8.6 weeks for IVR and 16 weeks for IVB [27]. In another study in which patient outcomes of IVR and IVA were compared, the recurrence rate of IVR, at 48.1%, was greater than that of IVA (13.9%), and recurrence occurred later in the case of IVA (mean time to recurrence: 14.2 weeks, IVA vs. 8.2 weeks, IVR) [28]. These findings indicate that IVR may have a shorter duration of action and a higher likelihood of recurrence compared to other agents.

Despite its higher recurrence tendency, IVR is often favored for its safety profile. IVB and IVA have been associated with prolonged systemic VEGF suppression and longer systemic half-life: IVB, systemic half-life approximately 18.7 to 20 days, with plasma VEGF suppression lasting up to 30 to 42 days [29,30]; IVA, systemic half-life approximately 5 to 6 days, with VEGF suppression observed up to 22 to 35 days [30,31], and potential adverse effects on neurodevelopment and postnatal growth [32,33]. In contrast, IVR has a short systemic half-life (approximately 1.5 to 2 h) [34] and a more limited impact on circulating VEGF levels, with suppression lasting approximately 3.7 to 7 weeks [30]. This pharmacokinetic advantage may translate into a lower systemic risk, making IVR a safer option, especially in fragile preterm infants.

Ideally, treatment for retinopathy of prematurity (ROP) should be completed without the need for laser photocoagulation. However, all currently available anti-VEGF monotherapies show a certain proportion of disease recurrence, and no agent has yet achieved both robust efficacy in ROP and minimal systemic impact. Moreover, late reactivation after anti-VEGF therapy has been reported up to 69 weeks PMA, necessitating rigorous and long-term follow-up [35]. Furthermore, no consensus exists on the optimal management of recurrent cases—whether additional anti-VEGF injections or laser photocoagulation (LPC) are preferable. Because of the unpredictable timing and nature of recurrence in ROP, missed or delayed follow-up can lead to rapid disease progression. Recurrence most often occurs within several weeks after the initial treatment, requiring frequent short-term monitoring, which is difficult to sustain over extended periods. Previous reports have also demonstrated a higher recurrence tendency after anti-VEGF therapy than after laser treatment, reaffirming the need for prolonged surveillance following anti-VEGF therapy [18].

In urban centers or facilities with well-established follow-up systems, anti-VEGF monotherapy may be able to yield favorable outcomes under strict and continuous monitoring. Furthermore, recent advances in telemedicine have shown promise in improving the quality of screening and follow-up in underserved regions while reducing travel burdens for both patients and caregivers [36]. Continued development and implementation of such systems are expected to further optimize ROP management in diverse clinical settings. In contrast, in rural or peripheral regions, barriers such as long travel distances, shortages of specialists, and insufficient parental education hinder initial screening and consistent follow-up, leading to a certain proportion of loss-to-follow-up cases [37]. Considering these healthcare access constraints, rather than relying solely on anti-VEGF monotherapy, it is crucial to determine the most appropriate strategy for completing treatment while ensuring favorable visual outcomes. In particular, in remote areas or in facilities with limited follow-up capacity—even in urban settings—a planned combination of laser treatment following initial anti-VEGF therapy may be a rational approach to reduce the risk of recurrence or reactivation and to decrease dependence on continuous follow-up.

In the present study, we examined the effect of the two-step treatment of anti-VEGF therapy and LPC. This combined treatment significantly reduced the number of laser spots required compared to LPC monotherapy. In studies by Bhat et al., intravitreal bevacizumab was used followed by deferred LPC at a mean interval of 8.2 weeks [38]. The follow-up period was 6 months, during which no recurrence was observed. Importantly, the number of laser spots and the area of laser application were significantly reduced in the deferred laser group compared to laser monotherapy. The authors concluded that this two-step approach allowed for physiological vascularization during the anti-VEGF window, reducing the need for extensive laser treatment and achieving favorable anatomical and refractive outcomes [38]. Similarly, Gangwe et al. evaluated IVR followed by deferred LPC at a median of 4 weeks, with no recurrence during a median follow-up of 6 months [39]. The authors reported that, compared to conventional LPC monotherapy, this two-step approach significantly reduced the number of laser spots and the area of retina requiring ablation. The results were attributed to progressive peripheral vascularization during the anti-VEGF efficacy window; the authors emphasized the potential of this approach to limit laser-induced complications and support long-term visual development [39]. These results, along with our findings, support the utility of a deferred, second-step LPC after anti-VEGF injection as a safe and effective strategy, particularly when accompanied by careful monitoring of retinal vascularization.

In our analysis of the dataset, we detected a temporal trend in disease severity. In the zone classification of ROP, the two-step treatment group had a significantly higher proportion of Zone 1 cases, on the basis of which vascular development was considered to be delayed compared to the LPC monotherapy group. Furthermore, GA was significantly shorter in the two-step treatment group compared to the LPC monotherapy group. In previous studies, investigators noted that the shorter the GA, the higher the incidence of ROP [40,41] and the greater the severity [42,43]. In Japan, it has also been reported that due to increases in advanced maternal age, in vitro fertilization (IVF) and other assisted reproductive technologies, multifetal pregnancies are becoming more common. Combined with advances in neonatal care, there has been an increase in early cesarean deliveries and preterm birth interventions, leading to an increasing number of births at shorter GA [44]. This shift has increased survival of extremely premature infants, which has paradoxically led to an increase in severe ROP cases in extremely premature infants. A retrospective study involving 298 eyes of 149 infants demonstrated that eyes with more severe disease—particularly those in Zone I or diagnosed with aggressive ROP (A-ROP)—had significantly higher recurrence rates after intravitreal ranibizumab monotherapy. Specifically, the recurrence rate was 19.1% in Zone I compared to 10.1% in Zone II, and 49.6% in A-ROP compared to only 4.1% in non-A-ROP cases [23]. These findings suggest that as ROP severity increases, monotherapy with anti-VEGF agents becomes less effective, often necessitating additional interventions—such as LPC, which underscores the importance of assessing and adopting combination therapies.

This study has several limitations. It is a retrospective study with a relatively small sample size and limited follow-up duration. Therefore, long-term recurrence rates could not be adequately evaluated. In addition, patients were not followed until an age at which visual acuity and visual fields could be assessed, and thus visual function outcomes were not examined. Furthermore, regarding the timing of laser photocoagulation (LPC) after anti-VEGF therapy, we were unable to determine whether 4 weeks was optimal or whether a longer interval, such as 6 or 8 weeks, might have been appropriate; therefore, a comparative assessment of the optimal interval could not be performed. Moreover, the extent of avascular retina after anti-VEGF therapy was not quantified, leaving the relationship between reduced laser application and actual retinal vascular development uncertain. Despite these limitations, our findings support the use of a two-step treatment strategy to effectively manage ROP.

In conclusion, combining anti-VEGF therapy with LPC significantly reduced the laser burden without compromising treatment outcomes. This two-step treatment offers an effective and promising treatment strategy for the management of ROP, particularly in severe cases and in settings where recurrence risk and long-term follow-up compliance are critical concerns.

## Figures and Tables

**Table 1:** Biographic data of the study group.

	Total	Group				A vs. B *p*-Value
		A	B	B1	B2	
Number of eyes	142	98	44	28	16	
Birth weight (g)	699.2 ± 175.02	720.3 ± 188.39	652.3 ± 133.49	652.9 ± 146.2	651.1 ± 112.3	0.0504
Gestational age (weeks)	25.6 ± 1.69	26.0 ± 1.67	25.0 ± 1.62	24.9 ± 1.23	25.3 ± 2.17	0.0023
Postmenstrual age (weeks)	33.5 ± 2.15	33.5 ± 2.09	33.4 ± 2.11	32.8 ± 1.11	34.6 ± 5.65	0.96

ROP stage						
Zone 2, Stage 2+	78	64	14	6	8	0.0001
Zone 2, Stage 3+	4	3	0	0	0	
Zone 1, Stage 3	2	1	1	1	0	
Zone 1, Stage 1+	15	8	7	7	0	
Zone 1, Stage 2+	26	8	19	12	7	
Zone 1, Stage 3+	17	14	3	2	1	

Number of LPC spots	487.6 ± 330.20	583.0 ± 350.72	274.9 ± 124.77	300.9 ± 115.5	229.5± 130.9	<0.0001
Number of LPC sessions	1.6 ± 1.12	1.8 ± 1.28	1.2 ± 0.45	1.1 ± 0.31	1.3 ± 0.60	0.0003
Vitrectomy, yes/no (eyes)	3/139	3/95	0/44	0/28	0/16	0.59

ROP, retinopathy of prematurity; LPC, laser photocoagulation.

**Table 2:** Multivariate analysis of factors associated with laser photocoagulation spot number.

Variable	Estimate	Standard Error	*t* Value	*p* Value
Intercept	1928.8795	421.9832	4.57	<0.0001 *
Birth weight	−0.5483	0.1709	−3.21	0.0017 *
Gestational age	−4.9197	2.8072	−1.75	0.082
Postmenstrual age	−1.0427	1.7557	−0.59	0.5536
Zone (1 vs. 2)	53.4438	25.4448	2.10	0.0376 *
Treatment group (A vs. B)	−206.0733	25.4162	−8.11	<0.0001 *

* Statistically significant values (*p* < 0.05).

**Table 3:** Number of patients in each group according to zone classification.

	Group A	Group B	*p*-Value
Zone 1	31	30	<0.0001
Zone 2	67	14	

## Data Availability

The datasets generated and/or analyzed in the current study are available from H. Imai, the corresponding author, upon reasonable request.

## Abbreviations

The following abbreviations are used in this manuscript:

ROP	Retinopathy of prematurity
A-ROP	Aggressive retinopathy of prematurity
VEGF	Vascular endothelial growth factor
LPC	Laser photocoagulation
IVR	Intravitreal ranibizumab
IVB	Intravitreal bevacizumab
PMA	Postmenstrual age
GA	Gestational age
BW	Birth weight
